# Coming Home to the Fire: Community, Belonging, and Justice-Centered Telehealth for Transmasculine Aging Adults

**DOI:** 10.3390/healthcare14121697

**Published:** 2026-06-13

**Authors:** Braveheart Gillani, Rem Martin, Kate Freeman, Brenda Mathias, Augustus Klein

**Affiliations:** 1 College of Health and Human Services, School of Social Work, Violence Prevention Center, University of North Carolina, Charlotte, NC 28223, USA; braveheart@charlotte.edu (B.G.); kfreem48@charlotte.edu (K.F.); 2 Independent Researcher, Charlotte, NC 28223, USA; emma.jmartin0@gmail.com; 3 School of Social Work, University of Memphis, Memphis, TN 38152, USA; blmthas1@memphis.edu; 4 College of Integrated Health Sciences, University at Albany, State University of New York, 1400 Washington Ave., Albany, NY 12222, USA

**Keywords:** telehealth, transgender older adults, qualitative research, ethics of care, social connectedness, equity in healthcare, gender-affirming care, person-centered practice

## Abstract

**Highlights:**

**What are the main findings?**

- Transgender older adults still perform unpaid Trans 101 education at virtually every encounter.
- Digital health infrastructure systematically misgenders transgender patients through binary EHR architecture, producing measurable clinical harms, including portal abandonment, avoidance, and missed diagnoses.
- Healthcare access is politically contingent. A single election can revoke insurance coverage, and clinical decisions are shaped by providers managing political risk rather than patient need.
- Peer community networks are the primary source of clinically relevant knowledge for this population, filling gaps that formal medicine has failed to address for aging transmasculine bodies.

**What are the implications of the main findings?**

- Telehealth competency in transgender aging health must be a mandatory condition of provider access, not an optional training.
- Gender-affirming design must be a non-negotiable baseline for all digital health infrastructure, including EHRs, portals, and insurance platforms.
- Coverage protections for gender-affirming care must be structurally insulated from political volatility to make telehealth equity durable.
- Telehealth must be redesigned as a relational ecosystem that formally integrates peer knowledge and community connection, and not just a platform for one-on-one clinical transactions.
- The clinical knowledge gaps specific to aging transmasculine bodies require dedicated investment in longitudinal research; telehealth cannot deliver competent care for this population until that evidence base exists.

**Abstract:**

**Background:** Telehealth is increasingly positioned as a solution for healthcare access among older adults; yet for transgender older adults, its application remains undertheorized, inconsistently implemented, and frequently reductive. Structural barriers, including provider incompetence, administrative misgendering, insurance precarity, and the clinical invisibility of aging transmasculine bodies, shape this population’s relationship to telehealth in ways that existing frameworks have not adequately addressed. **Objective:** This study examines the structural conditions shaping transmasculine and gender-nonconforming older adults’ engagement with healthcare and telehealth, and centers their visions for transformed, justice-oriented virtual care. **Methods:** Four semi-structured focus groups (n = 14 transmasculine and gender-nonconforming older adults, ages 40–67) were conducted via Zoom in June 2024 and analyzed using Braun and Clarke’s reflexive thematic analysis. The study was designed according to community-based participatory research (CBPR) principles. This study followed the Consolidated Criteria for Reporting Qualitative Research guidelines to ensure methodological transparency in reporting. **Results:** Analysis yielded five themes: (1) the provider competency crisis; (2) administrative violence and the architecture of misgendering; (3) insurance, politics, and the precarity of access; (4) the aging transmasculine body as uncharted clinical territory; and (5) participants’ collective vision for relational, community-centered care. **Conclusions:** We introduce the Campfire Model of Relational Telehealth, a conceptual framework comprising five empirically derived pillars: gathering, warmth, collective knowledge, safety, and accountability. The model argues that telehealth must move beyond transactional encounters toward a relational ecosystem of care grounded in justice, belonging, and structural transformation. We conclude with a call to action for providers, policymakers, and researchers to dismantle structural barriers and advance telehealth that cultivates dignity, belonging, and equity.

## 1. Introduction

The global population is aging at an unprecedented rate. By 2050, persons aged 60 or older will comprise 22% of the global population, with those aged 80 or older projected to triple during the same period [1]. In response to this demographic shift and concurrent pressures on healthcare systems, telehealth has emerged as a proposed solution: positioned to increase access, reduce costs, and enable aging-in-place [2]. Yet this apparent universalism obscures a complex reality. Telehealth is neither neutral nor equally beneficial across all populations, and its rapid expansion has outpaced systematic examination of who benefits, who is excluded, and who is harmed.

Lesbian, gay, bisexual, transgender, and queer (LGBTQ+) older adults have been substantially left out of aging and gerontological research and clinical practice. The “Aging with Pride” longitudinal research program documents stark health inequities: transgender older adults report higher rates of disability, depression, poverty, and chronic disease compared to their cisgender and heterosexual peers [3,4]. These disparities are not inevitable outcomes of aging or gender identity. They are the accumulated consequences of lifetime exposure to discrimination, stigma, healthcare avoidance, economic marginalization, and structural exclusion [4]. Transgender adults have nearly twice the rate of disability of their cisgender counterparts, a gap that widens with age: a 27 percent chance of disability at age twenty rises to 39 percent by age fifty-five [5]. These trajectories suggest that the health profiles typically associated with older adulthood arrive earlier in transgender lives. The disability gap, the accumulation of deferred care, and the chronic physiological burden of minority stress do not wait until age 65 to manifest; for many transgender people, their health consequences are measurable by midlife [5,6,7]. Defining aging as beginning at 40 for this population is not an arbitrary methodological choice but a response to what the epidemiological evidence actually shows.

Transgender older adults experience what scholars term multiple jeopardies: the simultaneous and multiplicative effects of ageism, transphobia, and systemic inequality [6,7]. Healthcare avoidance is pervasive. Anticipated discrimination drives many transgender people away from clinical settings altogether, creating delayed diagnoses and compounding health conditions [8]. These dynamics of cumulative disadvantage and healthcare avoidance are introduced here as the empirical foundation for this study and developed in full theoretical detail in Section 2. The present analysis proceeds from the premise that these patterns are structural rather than incidental, and that telehealth interventions designed without accounting for them will reproduce rather than resolve existing inequities. For older transgender adults, these barriers are amplified by the accumulated weight of a lifetime of medical trauma, institutional betrayal, and the erosion of community spaces that historically provided mutual care, education, and solidarity. The impact is not merely statistical; it is a lived reality that shapes every decision about whether to seek care at all [9].

The emergence of telehealth as a dominant care modality has introduced new possibilities and new risks for transgender older adults. As the theoretical framework in Section 2 elaborates, the ways telehealth may reproduce or exacerbate existing inequalities have been documented in the recent literature; what has not been examined is how this dynamic is lived by transmasculine and gender-nonconforming older adults specifically. During the COVID-19 pandemic, telehealth adoption accelerated dramatically, with utilization increasing by over 38-fold relative to pre-pandemic levels [2]. For some transgender individuals, particularly those in rural areas or those lacking access to affirming local providers, telehealth expanded access to gender-affirming hormones, mental health support, and primary care [10]. Telehealth for trans and gender diverse communities showed early promise, with evidence that virtual platforms could reduce provider discrimination and geographic barriers [11]. However, these gains were distributed unevenly. Electronic health record (EHR) systems built on binary gender architectures, intake forms demanding male or female categorization, and algorithms designed for cisgender bodies systematically misgendered transgender patients and generated administrative friction regardless of the platform [12].

For transgender older adults specifically, the telehealth evidence base is nearly nonexistent. A 2017 scoping review found transgender older adults almost entirely absent from telehealth research literature [13]. This absence is not incidental. When marginalized populations are excluded from research, their needs become invisible, and systems designed without their input perpetuate existing inequities at scale. Understanding past experiences of suicidal ideation and trauma in the life narratives of transgender older adults reveals how cumulative healthcare failure and provider hostility create lasting psychological harm [14]. Multilevel factors affecting health equity for this population include provider bias, institutional policy failures, insurance gatekeeping, and state-level political threats [15]. Research on telehealth that fails to center these dynamics will produce frameworks that replicate the harms they claim to address.

Although this paper engages the broader literature on transgender older adults, the focus groups recruited specifically transmasculine and gender-nonconforming older adults, and this focus warrants explicit justification. Transmasculine people aging on long-term testosterone navigate a largely uncharted clinical terrain, including cardiovascular changes, bone density shifts, and hormonal dynamics in aging bodies, that differs substantively from the concerns of other transgender subgroups and that existing literature has almost entirely overlooked. Second, transmasculine older adults occupy a particular structural position within healthcare systems: they are often visually gender-congruent in ways that render their transgender status invisible unless disclosed, creating distinct patterns of administrative erasure and provider ignorance. Third, community-based participatory research principles guided participant recruitment; the transmasculine and gender-nonconforming communities whose networks made this study possible are the communities whose experiences it centers. Findings speak most directly to transmasculine and gender-nonconforming older adults and offer transferable, rather than universal, insights for transgender populations more broadly. This manuscript centers on the lived experiences of transgender older adults themselves. Through four semi-structured focus groups conducted in June 2024 with 14 transmasculine and gender-nonconforming older adults, we examine the structural conditions that shape this population’s engagement with healthcare and, by extension, with telehealth. Before asking what telehealth can offer, this study asks what participants actually live with: provider incompetence, administrative violence, insurance precarity, and clinical invisibility. These are the conditions a telehealth system encounters when it reaches this population, and any serious analysis of telehealth equity must account for them. The study then turns to how participants envision a transformed system grounded in justice, connection, and affirmation. Healthcare avoidance due to anticipated discrimination is not a psychological quirk but a rational response to systems that cause harm [8]. Our inquiry begins from this premise: that the problem is not transgender older adults’ reluctance to engage with telehealth; the problem is a telehealth system not designed for them.

We performed a review of literature on telehealth for older adults, telehealth for LGBTQ+ populations, structural barriers in transgender healthcare, and transgender aging; a presentation of three interconnected theoretical frameworks; a description of methods; findings organized around five major themes; discussion; and a conclusion with recommendations and a call to action. Participants in this study were not merely research subjects; they are experts whose knowledge constitutes the intellectual foundation of this work.

This study adopts an age threshold of 40 or older, rather than the conventional gerontological threshold of 65 or older. This departure is grounded in cumulative disadvantage theory [16,17]: the mechanisms of structural exclusion specific to transgender lives, including employment discrimination, repeated healthcare avoidance, family estrangement, and the chronic physiological burden of minority stress [6,7] begin accruing in adolescence and compound across decades, such that many transgender people carry a health burden by midlife that reflects decades of structural exclusion [5]. The full methodological rationale for this threshold is provided in Section 3.2.

Despite growing attention to telehealth equity and LGBTQ+ health, a critical gap persists: there is almost no research that centers the experiences of transmasculine and gender-nonconforming older adults in relation to telehealth, or that grounds telehealth design recommendations in this community’s own visions for care [13]. This study addresses that gap directly. The aim of this study is twofold: first, to examine the structural conditions that shape transmasculine and gender-nonconforming older adults’ engagement with healthcare and, by extension, with telehealth; and second, to center participants’ own articulations of what transformed, affirming, justice-oriented care could look like. In doing so, this study contributes a community-derived conceptual framework, the Campfire Model of Relational Telehealth, to the fields of gerontology, LGBTQ+ health, and digital health equity.

## 2. Background and Theoretical Framework

### 2.1. Telehealth for Older Adults

Telehealth adoption among older adults has expanded substantially over the past decade. The COVID-19 pandemic dramatically accelerated this shift, with telehealth utilization rising more than 38-fold compared to pre-pandemic levels [2]. For older adults, telehealth offers meaningful benefits: reduced transportation burden, decreased psychological stress related to travel, improved continuity of care for chronic conditions, and expanded access to specialist services not available locally [2]. It reduces anxiety and depression among older adult users and improves medication adherence, suggesting real clinical benefit when access is achieved [2].

Despite these benefits, barriers to telehealth adoption remain substantial and systematically skewed toward already-disadvantaged populations. Technical literacy gaps affect approximately 17 percent of older adults, and cost represents a barrier for roughly 8 percent [2]. More critically, digital equity gaps rooted in systemic inequalities of race, income, geography, and disability mean that telehealth access is deeply unequal. Rural older adults, older adults of color, and those with lower incomes face disproportionate barriers to broadband access, device ownership, and digital literacy [2]. A 2025 review examining equitable telehealth for older adults emphasizes that scaling telehealth without addressing these structural inequities effectively scales up exclusion [13].

Social connectedness has emerged as a critical dimension of telehealth’s potential impact on older adult health. Loneliness and social isolation carry measurable, well-documented health risks across the lifespan, and older adults face an elevated risk of isolation due to mobility limitations, geographic distance from family, and loss of peers [18,19]. Successful telehealth interventions for older adults integrate technology with genuine relational connections rather than positioning technology as a substitute for human relationships [20]. This insight is foundational to understanding what telehealth can and cannot do for transgender older adults, for whom social connection is not merely a wellness consideration but a survival and community resource.

### 2.2. Telehealth for LGBTQ+ and Transgender Populations

Building on the structural critique introduced above, the following reviews what the evidence base shows specifically for LGBTQ+ and transgender populations. The expansion of telehealth during COVID-19 initially appeared to hold particular promise for LGBTQ+ populations, particularly those in rural areas with limited access to affirming providers. Evidence on telehealth for trans and gender diverse communities suggested that virtual platforms could reduce provider discrimination, expand geographic access to gender-affirming care, and reduce the psychological burden of navigating potentially hostile clinical spaces [11]. The pandemic disproportionately challenged the mental health of transgender and gender diverse populations, and telehealth interventions focused on gender affirmation and mental health were found to be desired and acceptable by the community members [11].

However, structural failures embedded in telehealth systems constrained these gains. EHR systems built on binary gender frameworks consistently misgendered transgender patients through automated communications, appointment reminders, and clinical documentation [12]. receiving the least benefit and remaining most invisible to systems claiming to serve all. Recent studies examining telehealth for gender-diverse youth identify barriers with direct relevance to older adults: lack of visual privacy in home-based settings, concerns about confidentiality when family members are present, and the dangerous assumption that telehealth eliminates the need for provider competency and affirming practice [21]. For older transgender adults, these barriers are compounded by age-related factors, including a greater likelihood of disability, lower digital literacy, and accumulated healthcare trauma that makes any clinical encounter, including virtual ones, a context requiring careful navigation and genuine trust-building [14].

### 2.3. Structural Barriers in Transgender Healthcare

The structural barriers documented in the literature take on specific dimensions for transmasculine and gender-nonconforming people, a point returned to throughout this paper. Structural barriers to transgender healthcare are pervasive, interlocking, and resistant to individual-level solutions. Foundational analysis of administrative violence identifies how harm is enacted not through overt refusal but through bureaucratic processes: the multiplication of paperwork, the demands for documentation that transgender people cannot produce, the misgendering embedded in administrative systems, and the prior authorization processes that deny access through delay [22]. Insurance systems, EHR design, intake forms, and institutional policies are all sites of administrative violence [22]. These systems do not require individual actors to intend harm; the harm is built into the architecture.

The scale of healthcare avoidance driven by these structural failures is documented across multiple national studies. The 2015 U.S. Transgender Survey found that 33 percent of respondents reported a negative experience with a healthcare provider, 23 percent reported not accessing needed care due to fear of discrimination, and 27 percent of transgender men specifically avoided healthcare due to anticipated mistreatment [9]. Healthcare avoidance due to anticipated discrimination among transgender people represents not a failure of individual courage but a rational response to systems that have demonstrated their capacity to cause harm [8]. Multi-level barriers spanning individual provider bias, institutional policy, insurance structures, and state legislation operate simultaneously and synergistically [15]. Research examining systemic transphobia and ongoing barriers to healthcare identifies how historical exclusion, clinical ignorance, and institutional indifference combine to create what amounts to organized abandonment of transgender patients [23]. The burden placed on transgender older adults to educate their providers about transgender health is not a minor inconvenience; it constitutes exploited, unpaid labor that compounds the burden of marginalization [24].

### 2.4. Transgender Aging and Cumulative Disadvantage

Transgender aging has only recently begun to receive dedicated research attention, and the evidence base remains thin relative to the complexity and urgency of the problem. Dannefer’s theory of cumulative advantage and disadvantage offers a foundational framework for understanding why transgender older adults arrive at later life with disproportionately compromised health [16]. The theory proposes that small initial inequalities, rather than excessive withdrawal of resources over time, diverge progressively through feedback loops that amplify advantage for those who begin with resources and compound disadvantage for those who begin without them. Applied to transgender aging, cumulative disadvantage operates through multiple interconnected mechanisms that span decades of a person’s life.

Ferraro and Shippee’s extension of Dannefer’s model emphasizes the concept of cumulative inequality as embodied disease: disadvantage accumulates not merely in social and economic domains but in the body itself, as chronic stress, deferred healthcare, environmental degradation, and repeated trauma inscribe themselves in the body [17]. For transgender people, this embodied cumulation is traceable across multiple life domains. Employment discrimination across decades reduces lifetime earnings and retirement savings, creating late-life poverty that constrains access to care [17]. Repeated healthcare avoidance due to anticipated discrimination produces compounded missed diagnoses [17]. Housing discrimination creates unstable physical environments that affect health trajectories across decades. Family estrangement removes the informal caregiver networks that typically sustain older adults through illness and aging [17]. Minority stress, the chronic physiological activation generated by stigma, prejudice, and discrimination, accumulates as allostatic load over decades [6,7].

Transgender adults have nearly twice the rate of disability of their cisgender counterparts across all age groups, a gap that reflects these decades of compounding disadvantage [5]. Research on health disparities among LGBTQ+ older adults using a structural competency approach confirms that these disparities cannot be addressed through cultural sensitivity training alone but require systemic transformation [25]. Understanding past experiences of suicidal ideation and behavior in transgender older adult life narratives reveals how cumulative healthcare failure, family rejection, economic precarity, and social isolation create trajectories that converge in later life crisis [14,26]. A 2025 study examining multilevel factors affecting health equity for transgender and gender diverse older adults identified that these intersecting vulnerabilities require multi-level interventions spanning individual, relational, organizational, and policy levels [15].

### 2.5. Theoretical Models

Ethics of care, developed through the work of Noddings (1984), Gilligan (1982), Tronto (1993, 2013), and Held (2006), begins from the premise that human life is fundamentally characterized by interdependence and that responding to need is a moral imperative [27,28,29,30,31,32]. Tronto identifies five phases of care: caring about, taking care of, care-giving, care-receiving, and caring with, insisting that care must be consistent with democratic values of equality and justice [28,29]. Crucially, the ethics of care demands attention to what Tronto calls “privileged irresponsibility”: how dominant groups systematically avoid accountability for care work [28]. Applied to telehealth, the ethics of care insists that questions of access and quality are also questions of justice. Noddings further emphasizes that genuine caring requires attentiveness: the sustained, responsive attention to the particular person before us, not a standardized protocol applied generically [27]. Fisher and Tronto define caring as “a species activity that includes everything that we do to maintain, continue, and repair our world so that we can live in it as well as possible” [30,31].

Person-centered practice, developed by McCormack and McCance (2010, 2017), offers a framework for healthcare that honors the whole person rather than reducing the individual to a diagnosis or condition [32,33,34]. The framework rests on four constructs: prerequisites, care environment, person-centered processes, and outcomes [33,34]. Personhood is understood not as a fixed attribute but as a relational achievement, something that must be actively recognized and supported by care systems [35]. Roe, 2021, extend person-centered practice explicitly to LGBTQ+ healthcare contexts, demonstrating that this requires understanding social contexts, values, histories of trauma, and specific health needs [36].

Social connectedness, understood as the subjective sense of belonging and meaningful connection to others, is a fundamental determinant of health across the lifespan. The evidence is unambiguous: loneliness and social isolation carry health risks equivalent to smoking 15 cigarettes per day, and these risks are particularly pronounced in later life [18,19]. Cornwell and Waite demonstrate that both objective social disconnectedness and subjective perceived isolation independently predict mortality, morbidity, and disability in older populations [37]. Lyyra and Heikkinen document that feelings of belonging and social participation are particularly protective health factors for older adults [38]. Community involvement and peer support serve as critical protective factors for transgender mental health [39]. A 2022 review found that technology-based interventions successfully reduced social isolation only when they integrated genuine relational connections rather than positioning technology as a substitute for human relationships [20].

These three theoretical frameworks—ethics of care, person-centered practice, and social connectedness—form an integrated analytical scaffold rather than operating independently. Each framework contributes a distinct lens to both the analysis and the development of the Campfire Model. Ethics of care informs the analytical attention to relational attentiveness and accountability, grounding the study’s critique of transactional telehealth and directly shaping the warmth and accountability pillars of the Campfire Model. Person-centered practice informs the analysis of how current healthcare systems fail to recognize transmasculine patients as whole persons, directly shaping the safety and warmth pillars. Social connectedness research grounds the study’s argument that peer community networks are not merely social supports but clinical health resources, directly shaping the gathering and collective knowledge pillars. Together, these frameworks provided both the lens through which focus group data were interpreted and the conceptual vocabulary through which participant visions were translated into a design-ready model. The Campfire Model, therefore, represents the synthesis of participant wisdom and theoretical frameworks, not participant data alone.

## 3. Methods

### 3.1. Study Design

This qualitative research employed a focus group design guided by reflexive thematic analysis [40,41,42,43,44]. This study followed the Consolidated Criteria for Reporting Qualitative Research (COREQ) guidelines to ensure methodological transparency in reporting. Focus groups were selected as the primary method because they create space for collective meaning-making, allowing participants to build on one another’s experiences, challenge one another’s framings, and generate insights that individual interviews would not surface. Reflexive thematic analysis, as articulated by Braun and Clarke (2006, 2021), emphasizes the researcher’s active role in constructing meaning from data rather than treating themes as naturally occurring entities awaiting discovery [44,45]. The study was designed according to community-based participatory research (CBPR) principles, recognizing transgender older adults as experts in their own experiences and establishing accountability to the community as a core research commitment [46]. Data collection occurred in June 2024. Telehealth emerged organically and recurrently across all four sessions as a theme of significant salience, raised by participants themselves in discussions of geographic barriers, provider availability, insurance instability, sexual health access, and relational care ideals.

### 3.2. Participants, Recruitment, and Focus Group Characteristics

Participants were recruited through purposive sampling, intentionally seeking individuals with lived experience as transgender older adults. Recruitment was conducted through multiple community-embedded channels to reach transmasculine and gender-nonconforming older adults across diverse geographic contexts. Specifically, recruitment occurred via: (1) LGBTQ+-affirming community organizations and advocacy groups, including those with programming specifically for older adults; (2) transgender-focused online forums, listservs, and social media communities; (3) provider referrals from clinicians known to serve transgender patients; (4) word-of-mouth through peer networks accessed via a community advisory board convened for this study; and (5) digital and physical flyers distributed through LGBTQ+ resource centers and social service agencies. Recruitment was designed to achieve geographic diversity, with deliberate outreach to rural and suburban participants given the study’s telehealth focus. The research team acknowledges a potential self-selection bias: participants recruited through community networks may be more socially engaged and institutionally connected than more isolated transgender older adults, whose experiences—potentially more acute—are underrepresented in this sample. Inclusion criteria were: (1) age 40 years or older; (2) identified as transgender; (3) sufficient English fluency to participate in group discussion. English fluency was required because focus groups were facilitated in English and no interpreter support or multilingual facilitation resources were available for this study. The research team acknowledges that this criterion constitutes a meaningful exclusion: non-English-speaking transgender older adults—for whom language access barriers compound existing healthcare inequities—were not eligible to participate.

Fourteen participants were enrolled across four focus groups. Of the 14 participants, the majority identified as transmasculine; several identified as gender-nonconforming or nonbinary with a transmasculine trajectory; and a small number held broader transgender identities that did not fit neatly within either category. The demographic composition of the sample shapes the interpretive scope of findings, and readers should consider how race, class, and geography may intersect with the barriers documented in Themes 1–4.

A note on sample scope and interpretive reach is essential. Throughout this manuscript, the term ‘transgender older adults’ is used when reviewing the broader literature. The focus groups, however, recruited specifically transmasculine and gender-nonconforming older adults, not the full range of transgender identities. The structural barriers documented in Themes 1–4 are grounded in the specific experiences of transmasculine and GNC people. While many of these barriers are shared across transgender subgroups, the particular clinical terrain of aging on testosterone (Theme 4) is specific to transmasculine people and should not be generalized to transgender women, nonbinary people with different hormone histories, or transgender people who have not pursued medical transition. Where the manuscript refers to the broader literature, we use ‘transgender older adults’; where findings are grounded in this study’s data, we use ‘transmasculine and gender-nonconforming older adults in this study.’

Participants ranged in age from their early 40 s to 67 years, with transition histories spanning from approximately 3 years to more than 30 years. Geographic diversity was maintained, with participants residing in urban, suburban, and rural areas.

The age threshold of 40 or older requires explicit justification in a study that uses the language of older adulthood. Standard gerontological convention sets that threshold at 65, and this study’s sample, which includes participants as young as their early 40 s and only three participants aged 65 or older, departs meaningfully from that convention. That departure is grounded in the theoretical framework of this paper and in the empirical evidence base on LGBTQ+ aging.

Dannefer’s theory of cumulative disadvantage, and Ferraro and Shippee’s extension of it as embodied disease [16,17], provide the conceptual foundation. Disadvantage does not accumulate on a schedule calibrated to cisgender norms. For transgender people, the mechanisms of cumulative disadvantage, which include employment discrimination, repeated healthcare avoidance, family estrangement, housing instability, and the chronic physiological burden of minority stress, begin accruing in adolescence and young adulthood and compound across decades [6,7,17]. By midlife, many transgender people carry a health burden that reflects decades of structural exclusion. The disability data are instructive: transgender adults have nearly twice the disability rate of cisgender counterparts across all age groups, and that gap is traceable not to age alone but to the cumulative weight of disadvantage [5]. LGBTQ+ aging scholars have therefore argued that the health profiles associated with older adulthood manifest at earlier chronological ages in this population, and that research designs calibrated to the 65+ threshold systematically exclude the cohort most likely to be living the consequences of lifelong minority stress [6,7].

This study adopts the 40+ threshold on that basis. Participants in their 40s and 50s who have been on testosterone for two or more decades and are navigating cardiovascular changes, bone density concerns, and the absence of clinical protocols for their bodies are, by any clinically meaningful measure, aging transgender adults. Restricting the sample to those 65 or older would have excluded precisely the participants whose embodied experiences are most relevant to the paper’s research questions. Of the 14 participants, 3 were aged 65 or older, and their experiences are included in full; the remaining participants represent the midlife-to-older-adult continuum that LGBTQ+ aging research has consistently identified as a population requiring dedicated attention [6,7,15].

The research team anticipated and actively managed the power dynamics specific to focus group methodology with a trauma-affected community. Facilitators received training in trauma-informed facilitation practice, with particular attention to: (1) managing dominant voices and creating space for quieter or more hesitant participants; (2) recognizing and responding to emotional distress; (3) supporting participants’ rights to pass, redirect, or withdraw from specific discussions without penalty; and (4) maintaining disclosure boundaries, with explicit instruction to participants at the outset of each session that disclosures were expected to remain within the group. A trauma-informed distress protocol was in place: facilitators were prepared to pause discussion, offer grounding exercises, and provide referrals to mental health support resources if participants became distressed. The research team acknowledges that focus group settings inherently carry the risk that more vocal participants may shape discourse in ways that silence more vulnerable voices. Facilitators actively monitored for this dynamic and used structured turn-taking and direct invitations to ensure all participants had opportunity to contribute.

This study combines a literature review with primary qualitative data, and the integration of these two components in the development of the Campfire Model warrants explicit description. The literature review (Section 2) was conducted a priori: theoretical frameworks were identified before data collection as a conceptual lens to guide analysis rather than as a source of themes. The qualitative focus group data (Section 3 and Section 4) were the primary generative source for the Campfire Model: the five pillars were derived inductively from participant data—specifically from Theme 5 and the structural realities documented in Themes 1–4—rather than deduced from the theoretical frameworks. Following theme development, the theoretical frameworks were applied post-analytically to situate participant visions within existing conceptual traditions. The integration logic is therefore: theory as lens for analysis, data as source of the model, theory as retrospective situating of the model. This reflects the study’s CBPR commitment to participant wisdom as the primary source of knowledge.

While the theoretical rationale for this threshold is well-grounded, the authors acknowledge the methodological and conceptual implications of this departure. Findings from this study are not directly comparable to gerontological research calibrated to the 65+ standard, and cross-study comparisons should account for this difference. The inclusion of participants in their early 40 s may mean that some findings reflect midlife concerns—employment, active parenting, workforce-related insurance—as much as late-life ones. Future research should test whether findings hold for exclusively older cohorts (65+) and explore how findings differ across the age spectrum represented here.

### 3.3. Data Collection

Four focus groups were conducted via Zoom videoconferencing during June 2024.The decision to use Zoom warrants explicit reflexive engagement given this manuscript’s central argument that telehealth platforms replicate structural inequities unless equity is designed in. Prior to enrollment, all prospective participants were assessed for adequate technological access: this included confirming access to a device with video capability, a stable internet connection, and sufficient digital literacy to navigate Zoom independently or with minimal support. All sessions were conducted in password-protected Zoom rooms with the waiting room feature enabled. Participants were encouraged to join from private spaces, and the facilitation team acknowledged at the outset of each session that home-based participation might carry privacy risks (e.g., the presence of household members) and invited participants to use pseudonyms if preferred. The research team acknowledges that despite these precautions, digital access inequities may have created differential conditions of participation—particularly for rural participants and those with lower digital literacy. Future research should build in more robust digital equity supports, including the option of telephone participation for those without reliable video access.

Each session lasted 60 to 90 min and was facilitated by two researchers with experience in trauma-informed, community-engaged qualitative research. Facilitators used a semi-structured interview guide that explored healthcare experiences, relationships with providers, social support systems, sexual health concerns, and experiences specific to aging in transgender bodies. All sessions were audio-recorded with participant consent and professionally transcribed. Transcripts were de-identified before analysis; all personally identifying information was removed.

### 3.4. Analytical Approach

Data saturation was not applied as a stopping criterion in this study, consistent with reflexive thematic analysis epistemology. Braun and Clarke [44,45] argue that saturation is a concept derived from grounded theory and phenomenology whose application to reflexive TA misaligns with the framework’s constructivist underpinnings: in reflexive TA, there is no fixed point at which ‘all’ themes have been discovered. Sample size was instead determined by the CBPR design: four focus groups were identified in advance as the appropriate structure for this community-engaged inquiry, balancing depth of engagement with participant burden. The four focus groups produced analytically rich data and the team determined that thematic depth was sufficient for the study’s aims.

Data analysis followed the six-phase approach to reflexive thematic analysis outlined by Braun and Clarke [44,45]. Following the reflexive thematic analysis framework [44,45], two members of the research team independently engaged in coding as a practice of reflexive dialogue rather than inter-rater reliability assessment. Braun and Clarke explicitly caution against applying reliability logic such as percentage agreement or Cohen’s kappa to reflexive TA, in which the goal is interpretive richness through comparison of divergent readings rather than coding convergence [47,48]. Accordingly, the parallel coding process was designed to surface interpretive differences and generate analytic conversation rather than produce a reliability coefficient. Where readings diverged, a third researcher facilitated structured memo-writing discussion through which divergences were examined as analytically productive. This approach reflects a hybrid reflexive-inductive orientation: inductive in that codes emerged from the data, and reflexive in that the research team’s positionality as community members was understood as shaping interpretation throughout.

Following coding, the nine preliminary domains were reviewed collectively by the full research team through a series of analytic meetings. Domains were examined for thematic coherence, conceptual distinctiveness, and salience within the dataset. Through iterative team discussion and structured memo writing [44,45], the domains were consolidated, refined, and reorganized into the five final themes presented in Section 4. This process was explicitly interpretive rather than mechanical: the movement from domains to themes involved active analytic decisions about which patterns best captured the data’s meaning in relation to the study’s research questions. Final themes were reviewed against the full dataset to confirm they were grounded across multiple participants and focus groups rather than driven by any single voice.

The research team engaged in sustained, open-minded reading of all four transcripts, generating reflective notes on meaning, resonance, and pattern. Line-by-line open coding produced 47 discrete codes organized into 9 preliminary domains spanning structural barriers, provider failures, trauma, aging, community, sexuality, telehealth implications, visions for care, and intersectionality. To enhance reliability, two members of the research team independently coded all four transcripts, with divergent segments adjudicated by a third researcher.

### 3.5. Ethics and Trustworthiness

This research received Institutional Review Board (IRB) approval (Protocol #2022-0408-Hunter). All procedures were conducted in accordance with the ethical principles of informed consent, voluntary participation, confidentiality, and minimization of harm.

Focus group methodology requires specific ethical safeguards beyond those applicable to individual interviews, and this study implemented the following. Intra-group confidentiality: at the start of each session, participants were explicitly asked to refrain from sharing others’ disclosures outside the group. The limitations of this commitment, i.e., the research team cannot enforce intra-group confidentiality beyond requesting it were acknowledged to participants, who were encouraged to share only what they felt comfortable having in a semi-public research context. Distress protocol: given the trauma-laden content of discussions (including healthcare violence, insurance loss mid-treatment, and clinical abandonment), the facilitation team maintained a distress response protocol throughout each session. This included the ability to pause discussion, offer grounding practices, and provide referrals to culturally competent mental health and crisis support services. A list of relevant support resources was shared with all participants at the conclusion of each session. Researcher wellbeing: members of the research team who identify as transgender or gender-nonconforming engaged in collective debriefs after each session to process vicarious and direct emotional responses to the material.

Researcher positionality shaped every phase of this study, and transparency about those positions is a methodological obligation within reflexive thematic analysis and CBPR. Several members of the research team identify as transgender or gender-nonconforming; some have personal experience of aging in transgender bodies and navigating the healthcare failures documented in this paper. Insider status facilitated participant trust, enabled more candid disclosures, and grounded the research team’s interpretive engagement in embodied as well as intellectual understanding. At the same time, insider status carries interpretive risks that the team actively managed: community members who identify with participants’ experiences may unconsciously amplify narratives that resonate with their own and bring advocacy commitments that shape analytic emphases. The team managed these dynamics through: (1) structured reflexive memos written after each coding session explicitly noting emotional responses and interpretive investments; (2) team analytic meetings that surfaced divergent readings and interrogated consensus; (3) member-checking, in which preliminary themes were shared with a community advisory group for feedback; and (4) inclusion of researchers with different positional relationships to the topic, whose perspectives provided analytic counterweight. Consistent with feminist standpoint theory [43,47] and CBPR principles [48,49], positionality is understood as a condition of rigorous, accountable knowledge production—not a threat to it—when subjected to systematic reflexive examination.

Participants provided verbal informed consent and were compensated for their time. Trustworthiness was established through credibility, transferability, dependability, and confirmability consistent with Lincoln and Guba’s framework [50]. Researcher positionality is explicitly acknowledged: members of the research team identify as members of the transgender and gender-nonconforming community, understood as an epistemological strength consistent with community-engaged research principles and feminist standpoint theory [46,47,50].

The following themes are derived from the accounts of 14 transmasculine and gender-nonconforming older adults in this study. Findings represent the experiences of this specific sample and should be interpreted as transferable rather than as universally representative of all transgender or transmasculine older adults.

## 4. Findings

Analysis of the four focus group transcripts yielded five major themes. Table A1 (see Appendix A) provides a summary of all five themes, their key sub-themes, exemplar quotes, and their correspondences to the five Campfire Model pillars; readers may find it useful to consult Table A1 alongside reading the theme descriptions that follow. Themes 1 through 4 document the structural conditions of healthcare that this population navigates on a daily basis: provider incompetence, administrative misgendering, insurance and political precarity, and the absence of clinical knowledge specific to aging transmasculine bodies. These themes establish what telehealth enters when it reaches this population. Theme 5 presents participants’ own visions for what care could look like if those conditions were addressed. The Campfire Model introduced in the Discussion is grounded in Theme 5 and takes the structural realities of Themes 1 through 4 as its starting point.

***Theme 1: The Provider Competency Crisis—Being Both Patient and Educator***

Among participants in this study, participants described a recurring dynamic in which they arrived at clinical encounters already knowing that they would need to educate their providers about basic transgender health.

One participant captured the cumulative exhaustion of this dynamic with particular clarity, describing how clinical encounters consistently returned to the earliest, most basic questions about transgender identity rather than addressing the complex, longitudinal health concerns of an aging body:

*“All of the things about transitioning that I have already dealt with and moved on from are still the things that people are most interested in asking me about. I have to give Trans 101 every time I walk into a new office. I just want someone to know what they’re doing.”*

*—Participant FD*

This statement captures the exhaustion of educational labor that transgender older adults absorb at every new clinical encounter. Providers trained on transgender health basics, when they are trained at all, receive education focused on early transition; they are not prepared for the person who has been on testosterone for decades and now needs cardiovascular monitoring, bone density screening, and aging-body hormone management. The phrase “moved on from” signals a developmental arc in transgender healthcare needs that clinical systems have failed to track. What this participant requires is not sensitivity training but genuine longitudinal clinical expertise, and the cost of its absence falls entirely on the patient.

For those who had managed to locate a competent provider, the relief was real but fragile, as another participant described when reflecting on what it meant to have a knowledgeable doctor in a rural setting:

*“I have a great doctor now in rural Northern California. She is competent. But I had to find her. And keeping her feels fragile. She could retire, or move, or the practice could close.”*

*—Participant HK*

This quote illustrates the precarity of competent care as a structural condition rather than a personal circumstance. Finding a knowledgeable provider requires extensive community research, word-of-mouth navigation, and often years of failed encounters; maintaining access to that provider is experienced as perpetually contingent on factors entirely outside the patient’s control. The fragility described here is not simply anxiety; it is an accurate assessment of a healthcare landscape in which affirming, competent providers are so scarce that losing one constitutes a genuine healthcare catastrophe, requiring the entire Trans 101 education process to begin again.

The failure of telehealth to resolve this dynamic was named directly by one participant who described a virtual encounter that reproduced the familiar pattern of provider ignorance despite the platform’s promise of expanded access:

*“I sat in that telehealth waiting room for forty-five minutes, and then they connected me to someone who had never heard of polycythemia in trans men. I knew more about my condition than they did. I could have been anywhere for that.”*

*—Participant DS*

The final sentence is analytically crucial: the participant has identified the precise failure mode of access-focused telehealth, in which geographic presence is replaced by virtual presence, but the fundamental barrier, which is provider ignorance, remains intact. Person-centered practice requires that providers approach encounters with the values, knowledge, and skills appropriate to the person before them [33]; routing transgender older adult patients through general provider pools without competency screening does not meet that standard, and the participant’s assessment of “I could have been anywhere for that” names the consequence with devastating economy.

Taken together, these accounts reveal a provider competency crisis that operates across every modality of care, in-person and virtual alike, and that compounds over time in ways the healthcare system has not begun to reckon with. The educational labor extracted from transgender older adults at each encounter is not a minor inconvenience but a cumulative tax on finite personal resources, which can tip the balance against seeking care at all. Until clinical training produces providers who arrive at encounters with genuine, longitudinal expertise in aging transmasculine health, expanded telehealth access will continue to deliver the same incompetence at greater speed and scale.

***Theme 2: Administrative Violence and the Architecture of Misgendering***

Every participant described encounters with administrative systems that misgendered them: electronic health records, appointment confirmation communications, insurance correspondence, lab result portals, and referral paperwork. This was not described as an occasional error but as systemic, consistent, and predictable. The pattern that emerged across all four groups points not to individual failures of attention but to a foundational design problem: healthcare administrative infrastructure built on binary-gender architectures that automatically produce misgendering at scale, without any human actor needing to intend harm.

One participant described the experience of requesting corrections to their EHR gender marker repeatedly, across multiple encounters, without any change occurring, illustrating how the system’s architecture absorbs and neutralizes individual attempts at correction:

*“The portal says ‘Female’ every single time. Every single appointment confirmation, every single lab result, every single reminder. I have asked them to change it four times. It never changes. After a while, you just feel like the system is telling you something.”*

*—Participant ER*

Administrative violence operates through bureaucratic architecture rather than through individual intention [22]; the EHR does not hate transgender patients, but its binary structure produces harm with the efficiency and consistency of intentional exclusion. The phrase “the system is telling you something” is analytically significant: the participant has correctly identified that repeated, uncorrectable misgendering communicates a message about belonging and recognition that transcends any single technical error, amounting to a sustained institutional statement that their identity does not exist within the system’s categories.

A second participant drew an explicit comparison between overt historical discrimination and the more diffuse but no less harmful experience of structural erasure, locating the current moment within a longer arc of navigating hostile institutions:

*“I transitioned in Indiana in the nineties. I know what overt hostility looks like. This is different. The form just doesn’t have a box for me. The system doesn’t know I exist. That erasure is its own kind of violence.”*

*—Participant KH*

This framing carries important theoretical weight: the participant is distinguishing between interpersonal hostility, which is visible, nameable, and contestable, and structural erasure, which operates through absence and neutrality and is therefore more difficult to challenge and easier for institutions to deny. Having navigated both, this participant identifies structural erasure as its own distinct form of violence—one that does not require a perpetrator with intent, only a system with categories that exclude. For telehealth platforms that automate communications at scale, this distinction is especially urgent: algorithmic misgendering is structural erasure delivered at speed, reaching patients through push notifications and automated emails before any human clinician enters the encounter. The cumulative weight of misgendering across multiple systems in a single week was described by another participant in terms that illuminated how accumulation transforms the meaning of individual events:

*“It said Ms..’ on the email. I know that’s small. But it was the third time that week from three different systems. By the third time, it’s not small anymore.”*

*—Participant GF*

The accumulation described here is the mechanism of cumulative disadvantage operating in a microcosm: each misgendering event might be dismissed as minor by an outside observer, but the accumulation of three such events in a single week, delivered through administrative systems designed to carry institutional authority, produces a qualitatively different experience that cannot be understood by adding the incidents together. What the participant is describing is not three small slights but the felt recognition that no healthcare system in their life has ever recognized their existence. This recognition arrives not through any single dramatic moment but through the relentless, automated repetition of the wrong name. The clinical consequences of this accumulation were named directly by a participant who described how sustained portal misgendering produced a decision to disengage from the platform entirely, with measurable health consequences:

*“I stopped using the portal for two months. I just couldn’t deal with it. And I missed a test result. That’s not a minor problem.”*

*—Participant WS*

This account completes the causal chain that runs through this entire theme: binary design produces misgendering; misgendering accumulates into a sustained experience of erasure; erasure produces rational avoidance; and avoidance produces missed diagnoses and interrupted care. The participant’s understated conclusion, “that’s not a minor problem,” names what the rest of the healthcare system has been slow to acknowledge: that administrative misgendering is not a dignity issue separate from clinical quality but a direct pathway to clinical deterioration. EHR systems, patient portals, telehealth platforms, and all digital health infrastructure must be designed with gender-affirmation as a baseline requirement, not an afterthought.

***Theme 3: Insurance, Politics, and the Precarity of Access***

Participants described insurance coverage for gender-affirming care as contingent, politically precarious, and subject to sudden elimination. What emerged across focus groups was not a picture of bureaucratic inconvenience but of healthcare access experienced as a fragile political achievement, always one electoral cycle or policy reversal away from disappearing. This precarity was not abstract or anticipated; participants described its concrete, bodily consequences in real time.

One participant situated their current insurance coverage within a specific political history, naming with precision exactly how tenuous that coverage remained:

*“I have insurance now because it started as a project at the Human Rights Commission, and a bunch of city employees got coverage. I think about that every election. One elected official could take that away.”*

*—Participant G.F*

This statement illustrates the political contingency of healthcare coverage for transgender people with a clarity that no policy document captures. A right or a stable institution does not secure the participant’s access to care; it is secured by a historical political victory that a single subsequent political defeat can reverse. This is the actualization of minority stress theory [6,7]: the chronic physiological burden of knowing that one’s healthcare depends on electoral outcomes is not metaphorical stress but a sustained physiological state with measurable health consequences, layered atop every other demand this population already carries.

The consequences of that precarity were not hypothetical for another participant, who described what happened when coverage was lost mid-process, during an active course of surgical care:

*“I started the process and then lost insurance coverage, and then got stuck halfway through a surgical process. That’s not just inconvenient. That’s dangerous.”*

*—Participant DS*

The word “dangerous” is clinically precise and should be understood as such. Interrupted surgical processes pose real medical risks: incomplete procedures, wound-care requirements that presuppose continued treatment, and physiological states that require ongoing clinical management to remain stable. Insurance loss that interrupts a surgical process is not an administrative setback that can be paused and resumed; it is iatrogenic harm produced at the intersection of insurance architecture and political volatility, and the body that absorbs it belongs entirely to the patient.

That transfer of institutional risk onto the patient’s body was described in even starker terms by a participant whose surgeon’s clinical recommendation was shaped not by the patient’s physiological needs but by the provider’s own assessment of political exposure:

*“The political hedging was around my ovaries. I was advised to retain them rather than pursue a hysterectomy because my surgeon said the political climate made him uncertain about covering follow-up care. He was managing his risk. With my body.”*

*—Participant SD*

This account is one of the most direct illustrations in the dataset of what person-centered practice failure looks like. Person-centered care demands that clinical decisions be grounded in the person’s values, needs, and expressed preferences [33,34]; this encounter inverts that framework entirely, grounding the clinical recommendation in the provider’s political risk calculation while the patient’s body becomes the site where that institutional risk is deposited and stored. The participant’s framing, “he was managing his risk, with my body,” names this inversion with a precision that no theoretical description improves upon.

A fourth participant described a different dimension of political and structural precarity, recounting the persistence required to navigate multiple telehealth encounters before accessing a standard preventive medication:

*“I finally got PrEP, but it took three telehealth visits to get it, and two of those were with people who seemed confused about why a trans man would need it. The third person just prescribed it without asking questions. That was telehealth at its best and worst in one story.”*

*—Participant D.S*

This account contains within it a compressed illustration of both telehealth’s potential and its failure mode. The eventual successful prescription demonstrates that telehealth can connect patients with competent, non-judgmental providers; the two preceding encounters demonstrate that it can just as efficiently scale provider confusion and implicit stigma across multiple sessions before that outcome is reached. The participant succeeded because they had the persistence and resources to attempt the encounter three times; the system offers no guarantee or accountability for patients who do not.

***Theme 4: The Aging Transmasculine Body—Uncharted Clinical Territory***

Participants with long transition histories described a shift in their primary healthcare concerns: from transition-focused care to aging-body care. After fifteen, twenty, or thirty years on gender affirming hormones, they are now navigating cardiovascular changes, bone density concerns, hormonal adjustments in aging bodies, and surgical aftercare complications in a clinical landscape that has almost no protocol for their bodies. What distinguished this theme from the provider competency concerns raised elsewhere was its specificity: participants were not asking for basic transgender awareness but for sophisticated, longitudinal clinical expertise that does not yet exist in any systematic form. One participant articulated this developmental shift with precision, identifying the gap between the questions their body now raises and the questions their provider is equipped to answer.

*“When I started my transition, I was concerned about what the hormones were going to do. Now I’m concerned about what fifteen years of hormones have done and what is still doing in a body that is also just aging. Those are very different questions, and my doctor doesn’t have answers to either one.”*

*—Participant DS*

*This statement locates the failure not in individual provider inadequacy alone but in the absence of a clinical evidence base that should exist and does not.* Cardiovascular changes, bone health effects of long-term hormone use, and aging-body hormone dynamics represent largely uncharted clinical territory in both gerontology and transgender health literature [26]. The participant is not requesting exceptional or specialized care; they are requesting what would be entirely routine longitudinal preventive medicine for any cisgender patient of comparable age, and the system cannot provide it because the research that would underpin clinical protocols was never conducted. The frustration of arriving at clinical encounters with more knowledge than the provider was expressed by another participant navigating a well-documented condition that providers nonetheless consistently failed to recognize.

*“I know I have polycythemia. I know it is not uncommon in trans men on long-term testosterone. What I don’t know is why my doctor acts like it’s the first time they’ve heard of this.”*

*—Participant, DF*

The participant’s knowledge of their own condition was acquired not through formal clinical guidance but through peer networks and community research, which is an epistemological inversion that runs throughout this dataset and speaks to the degree to which transgender older adults have been compelled to become self-taught experts in their own healthcare to survive encounters with under-prepared providers. That a condition as documentable as polycythemia in long-term testosterone users continues to surprise providers represents not individual ignorance but a systemic failure of medical education and continuing professional development to keep pace with an aging transgender population. A third participant named the mismatch between what providers offer and what aging transmasculine bodies actually require, distinguishing between the basic competency they are still being asked to supply and the longitudinal clinical expertise they actually need:

*“I’ve been on testosterone for twenty-one years, and I still have to explain the basics to every new provider. But the basics aren’t even what I need anymore. I need someone who knows about long-term cardiovascular effects, bone density, and what the research says about dose adjustments as you age.”*

*—Participant WS*

This account captures what might be called the double bind of aging transmasculine healthcare: participants are still being required to provide foundational transgender education while simultaneously needing care at a level of clinical sophistication that even a fully competent general provider would struggle to deliver. The clinical questions this participant raises about cardiovascular monitoring, bone density management, and evidence-based dose adjustment in aging bodies are precisely the questions for which no established protocol exists, leaving patients to navigate the frontier of aging transgender medicine without a guide, in a body the medical system does not yet understand. The absence of clinical answers was not merely a source of frustration but an active, ongoing source of physical risk, as the same participant described when recounting an eight-month wait for a response to a clinically urgent question about cardiovascular safety:

*“Should I be lowering my dose of testosterone as I age to guard against cardiovascular disease? I asked this question and was told they would research it and get back to me. That was eight months ago.”*

*—Participant WS*

The provider’s non-response is not merely a failure of follow-through; it reflects the genuine absence of an evidence base from which to draw a response. This participant is living in the gap between the clinical questions that aging in a transmasculine body raises and the research infrastructure needed to answer them—managing the physiological stakes of that gap in real time in their own body. The eight-month silence is the healthcare system’s most honest communication about how little it knows and how little urgency it attaches to finding out.

Themes 1 through 4 together describe the structural landscape that any telehealth intervention for this population must reckon with. When provider competency is absent, expanded virtual access delivers the same ignorance to more people more efficiently. When digital health infrastructure is built on binary architecture, telehealth platforms misgender patients before a clinician ever appears on screen. When insurance coverage is politically contingent, telehealth access is only as stable as the current legislative session. When no clinical evidence base exists for aging transmasculine bodies, telehealth connects patients to providers who cannot answer their most consequential questions. None of these problems are incidental to telehealth; they are the conditions within which telehealth currently operates. Theme 5 turns from this diagnosis to what participants themselves said care could look like if those conditions were changed.

***Theme 5: The Campfire Vision—Toward a Relational Telehealth Ecosystem***

Across all four groups, participants articulated visions for transformed care that shared a common architecture: warmth, collective knowledge, peer connection, safety from institutional harm, and a sense of being genuinely known. What distinguished this theme from the preceding four was its orientation: where earlier themes documented what current systems fail to provide, this theme captured what participants knew, from lived experience, that care could and should look like. These were not abstract aspirations but precise, practice-ready descriptions of a relational infrastructure that participants had already built for themselves in community spaces and were now asking healthcare systems to recognize, resource, and replicate.

The generative metaphor that gave this theme its shape was offered by one participant whose image of a campfire captured, in a single figure, everything that current telehealth is not:

*“What I want is something like a campfire. Somewhere you go to be warm, to be with people who know what you’re talking about without explanation, to share what you know, to feel safe. And you can come back. It’s always there.”*

*—Participant SD*

The campfire metaphor is analytically rich in ways that reward close attention. Fire as warmth: care that is responsive, present, and genuinely comforting rather than transactional. Fire as gathering: a community that assembles around a shared purpose, where the act of coming together is itself therapeutic. Fire as light in darkness: a resource that is navigable and findable even for those who are geographically isolated or institutionally abandoned. Each dimension of the metaphor maps precisely onto a failure mode of current telehealth for transgender older adults, and together they constitute a design brief as coherent as any produced through formal human-centered design methodology.

The inadequacy of formal clinical knowledge as a sole source of health guidance was named directly by another participant, who located their most reliable health information not in any clinical encounter but in the peer networks that telehealth has yet to recognize formally:

*“I found out more about my own health from people in online trans communities than I ever found out from any doctor. If telehealth could connect me to that, to people who have been through what I’m going through, that would be something real.”*

*—Participant DS*

This statement reframes the question of what telehealth is for: not merely a platform for delivering clinical services at a distance, but a potential infrastructure for connecting patients to the distributed, experiential expertise that currently circulates only in informal community spaces outside institutional reach. The phrase “that would be something real” is a pointed contrast with the participant’s experience of formal telehealth encounters, and it identifies peer connection not as a supplement to care but as a form of care that formal medicine has not earned the right to replace.

The specificity of that peer knowledge demand was articulated by a third participant, who drew a clear line between the credentialed knowledge of a clinician and the embodied knowledge of someone who has lived the same physiological experience:

*“I want to talk to someone who has been on T for twenty years and dealt with what I’m dealing with. Not just a doctor who read an article. Someone who knows.”*

*—Participant GC*

The distinction between academic knowledge and experiential knowledge carried here is not anti-scientific; it is epistemologically precise. This participant is not dismissing clinical expertise but rather identifying a category of knowledge that clinical training cannot produce: knowledge that comes from living in a specific body, navigating a specific set of systems, and accumulating decades of firsthand experience with conditions that the medical literature has yet to adequately study. This is a community-based participatory research epistemology applied directly to telehealth design [46]: the community possesses knowledge that the institution needs, and the institution has not yet built the structures to access it.

Safety within the campfire vision was defined not as the absence of acute harm but as something longitudinal and relational, as one participant described when articulating what it would mean to feel safe in a healthcare encounter, finally:

*“Safety means I don’t have to start from scratch every time. It means someone knows who I am, knows my history, and doesn’t make me explain myself. It means I can come back and it’s still safe.”*

*—Participant CX*

This definition of safety as continuity and recognition reframes what telehealth platforms need to provide in structural terms. Safety is not a feature to be added to an existing transactional platform; it is an entirely different orientation toward the patient encounter, one in which memory, history, and relationship are built into the architecture rather than incidental to it. The phrase “I can come back, and it’s still safe” points toward what is perhaps the most fundamental design requirement the campfire vision generates: a system that persists across time, accumulates knowledge of the person, and does not require them to re-establish their existence at every point of contact.

The synthesizing statement of this entire theme came from a participant whose single sentence compressed the full weight of the preceding five themes into one clear demand:

*“I want telehealth to be a place I can go that knows me. Not a waiting room, I have to convince every time.”*

*—Participant CX*

The contrast between “a place that knows me” and “a waiting room I have to convince every time” names, with economy and force, the entire distance between the telehealth that currently exists and the telehealth this population requires. The ethics of care framework insists that genuine caring requires sustained, responsive attention to the particular person [27]; the person-centered practice framework insists that personhood must be actively recognized and supported rather than assumed or ignored [33,34]; and the Campfire Model, derived from this dataset, insists that telehealth must be designed to know its patients longitudinally, warmly, and completely. These are not separate demands articulated through different intellectual traditions; they are the same demand, and it has been waiting to be met for a long time.

## 5. Discussion

**Figure 1:**
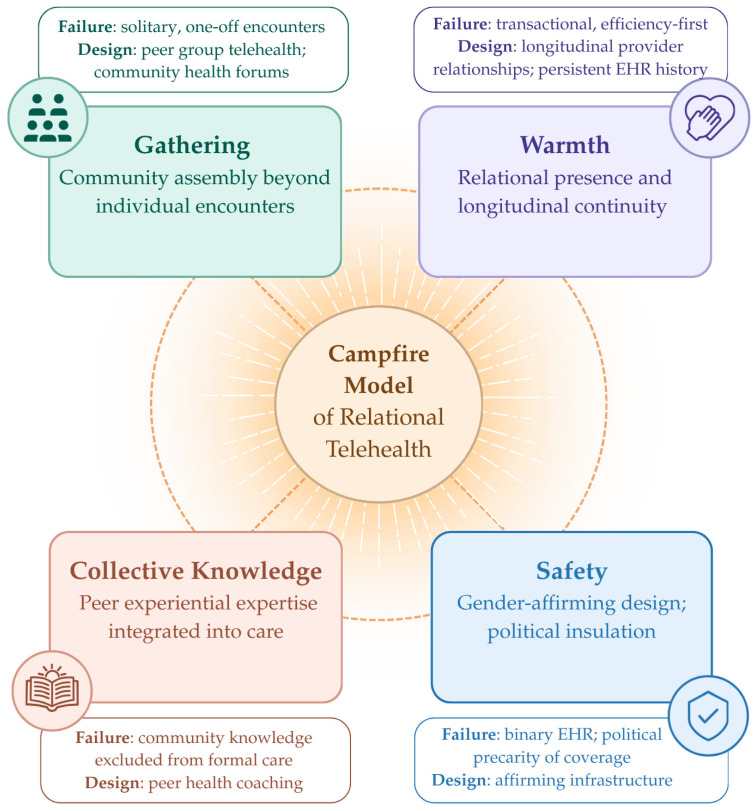
Campfire Model of Relational Telehealth.

The Campfire Model of Relational Telehealth is illustrated in Figure 1. Readers are encouraged to review the figure before proceeding, as it provides a visual map of the pillar-to-theme correspondences developed in the sections that follow.

The Campfire Model is empirically grounded: each of its five pillars is derived directly from the thematic analysis and reflects participant language. The mapping is as follows. The gathering pillar emerges from Theme 5, in which participants described the campfire metaphor and articulated the need for community assembly as a core healthcare function—captured most directly in Participant SD’s image of ‘somewhere you go to be warm, to be with people who know what you’re talking about.’ The warmth pillar also emerges from Theme 5, grounded in participants’ descriptions of relational continuity and longitudinal recognition—as Participant CX articulated: ‘I want telehealth to be a place that knows me.’ The collective knowledge pillar is derived from Themes 4 and 5 together: Theme 4 documents the absence of formal clinical knowledge for aging transmasculine bodies, and Theme 5 demonstrates that peer communities already hold distributed experiential expertise that formal medicine lacks, as Participant DS described: ‘I found out more about my own health from people in online trans communities than from any doctor.’ The safety pillar is derived from Themes 1 and 2: Theme 1 documents how provider incompetence produces avoidance, and Theme 2 demonstrates how binary EHR architecture and administrative misgendering accumulate into portal abandonment and missed diagnoses. The accountability pillar is derived from Themes 3 and 4: Theme 3 documents how insurance coverage is one electoral cycle away from elimination, and Theme 4 demonstrates that the clinical evidence base for aging transmasculine health does not yet exist.

The findings presented above converge on a clear and coherent vision, here formalized as the Campfire Model of Relational Telehealth. The model proposes five organizing pillars derived from participant data and grounded in the theoretical frameworks of this study. The first pillar, *gathering*, asserts that telehealth for transgender older adults must create space for community assembly rather than being restricted to isolated clinical encounters. The second pillar, *warmth*, holds that telehealth encounters must be relationally present rather than merely transactionally efficient. This requires that providers know their patients’ histories, that systems retain information across encounters, and that these interactions communicate genuine care. The third pillar, *collective knowledge*, posits that the expertise distributed across peer communities of transgender older adults is a health resource that telehealth must actively access and circulate, through peer health coaching, structured peer consultation, community-informed provider training, and the clinical legitimization of community-derived knowledge. The fourth pillar, *safety*, holds that telehealth for transgender older adults must be structurally designed to eliminate sources of administrative violence: this means gender-affirming EHR architecture, competent providers who arrive at encounters with longitudinal expertise rather than basic awareness, and care relationships designed to persist across time rather than reset at every visit.

The fifth pillar, *accountability*, addresses what the first four pillars require but cannot themselves produce. Themes 3 and 4 document conditions that no amount of relational redesign at the platform level will resolve: insurance coverage for gender-affirming care that is one electoral cycle away from elimination, and a clinical evidence base for aging transmasculine bodies that does not yet exist in any systematic form. A telehealth system grounded in gathering, warmth, collective knowledge, and safety can be built and still fail this population entirely if the coverage needed to access it evaporates under political pressure, or if the providers within it lack the research infrastructure to answer the clinical questions that participants are already living with. Accountability names the obligations that fall on institutions, insurers, researchers, and policymakers rather than on platforms or providers: structurally insulating gender-affirming coverage against political volatility, investing in longitudinal research on aging transmasculine health, and treating the clinical gaps documented in Theme 4 not as a niche concern but as a straightforward failure of medical research to keep pace with a population it has never adequately studied.

Taken together, the accounts documented in Theme 1 reveal a provider competency crisis that operates across every modality of care—in-person and virtual alike—and that compounds over time. The educational labor extracted from transmasculine older adults at each encounter is not a minor inconvenience but a cumulative tax on finite personal resources, which can tip the balance against seeking care at all. Until clinical training produces providers who arrive at encounters with genuine, longitudinal expertise in aging transmasculine health, expanded telehealth access will continue to deliver the same incompetence at greater speed and scale.

The Campfire Model makes a distinctive contribution to the existing telehealth equity and person-centered care studies, and its relationship to those frameworks warrants explicit articulation. The Campfire Model extends the structural analysis by moving beyond access (who can technically connect) to encounter quality (what happens when they do). Relational architecture, provider competency, administrative design, and peer knowledge integration are equally essential dimensions that structural telehealth equity frameworks have not yet systematically addressed. In relation to McCormack and McCance’s person-centered practice framework [33,34], the Campfire Model extends person-centered practice to the specific context of multiply marginalized older adults for whom ‘person-centered’ care requires not only attentiveness to the individual but structural dismantling of the administrative and political barriers that prevent that attentiveness from being possible. The Campfire Model operates simultaneously at three levels: (1) as a conceptual framework for researchers and theorists examining telehealth equity and transgender aging; (2) as clinical guidance for providers and healthcare organizations designing telehealth services for transmasculine and GNC older adults; and (3) as a system-level design tool for telehealth platform developers, EHR architects, and policymakers. The model is most immediately actionable at the clinical and organizational levels, where pillar-specific design changes can be implemented without waiting for policy-level transformation, while the accountability pillar names the structural and policy changes without which the other four pillars cannot be fully realized.

The age composition of this sample warrants direct engagement. Readers may question whether findings from participants as young as their early 40s genuinely represent older adulthood, given that standard gerontological frameworks set that threshold at 65. This paper’s use of a 40+ threshold is theoretically grounded in cumulative disadvantage theory [16,17], which documents that the health burden typically associated with older adulthood arrives measurably earlier in transgender lives due to decades of compounding structural disadvantage. Participants in their 40s and 50s who have been on testosterone for fifteen to thirty years and are navigating cardiovascular changes and bone density shifts are, by any clinically meaningful measure, experiencing the consequences of aging—regardless of chronological age. That said, this study acknowledges that findings from midlife participants may not fully capture the distinct experiences of those aged 65 and older, particularly with respect to generational differences in healthcare access and digital literacy. Future research should dedicate specific attention to the oldest segments of this population.

The distinction between findings directly derived from participant data, researcher interpretation, and broader policy extrapolation is maintained throughout this discussion. Participants’ accounts in this study suggest that EHR systems, patient portals, appointment reminders, and insurance verification processes are not neutral technological infrastructure but administrative architectures that yield differential effects across specific populations. Consistent, recurrent, multisystem misgendering reported by participants in this study indicates a foundational design failure rather than mere individual technical errors. Based on these findings, we interpret gender-affirming design as a clinical prerequisite rather than an accommodation. These data, in conjunction with existing literature, support the policy argument that gender-affirming design must be established as a foundational standard for all digital health infrastructure—though the authors acknowledge this claim extends beyond the scope of this dataset alone.

The EHR systems, patient portals, appointment reminders, and insurance verification processes described by participants are not neutral technological infrastructure; they are administrative architectures that yield differential effects across specific populations. Consistent, recurrent, multisystem misgendering reported by participants across all four groups indicates a foundational design failure, rather than mere individual technical errors. Telehealth equity for transgender older adults requires that a gender-affirming design be established as a foundational standard, not relegated to the status of an accommodation or edge case.

The ethics of care framework holds that genuine care requires attentiveness to the particular person rather than the application of generic protocols [27], while the person-centered practice framework insists that care must honor the whole person, not simply the presenting diagnosis [33,34]. Both frameworks are fundamentally undermined by the current transactional model of telehealth, in which efficiency, throughput, and documentation standards shape encounter design at the expense of relational presence and whole-person recognition. The Campfire Model addresses this gap by translating participants’ descriptions of effective care, i.e., being known, being safe, not having to recount their history from scratch, into a design framework that telehealth systems can implement.

The finding that participants derive more health-relevant knowledge from peer community networks than from formal clinical encounters challenges the traditional architecture of health knowledge production. CBPR principles demand that this knowledge be recognized and incorporated [48]. Consequently, telehealth designs that establish formal structures for the circulation of peer health knowledge– such as peer health coaching, moderated community health forums, and community-to-provider knowledge translation– would begin to address this gap.

The findings of this study support a specific set of policy and practice recommendations organized around the five pillars of the Campfire Model. In support of gathering and collective knowledge, telehealth platform developers must engage transgender older adult communities as co-designers from the outset, and formal structures for peer health coaching and community-to-provider knowledge translation must be built into telehealth architecture rather than left to informal networks. In support of warmth and safety, provider training requirements must include transgender health competency with specific attention to aging transmasculine bodies, long-term hormone management, and trauma-informed relational practice; and healthcare institutions must mandate gender-affirming design across all digital health infrastructure, including EHR systems, patient portals, appointment systems, and insurance communication platforms. In support of accountability, research funders must prioritize longitudinal studies of transgender aging so that the clinical knowledge gaps documented in this study are addressed systematically rather than left to individual providers to navigate, and insurance systems must provide stable coverage for gender-affirming care that is insulated from political volatility. These are not incremental improvements. They are structural obligations.

## 6. Limitations

This research has several important limitations. The sample of 14 participants across four focus groups, while appropriate for qualitative inquiry, limits the breadth of experiences captured. The sample spans a wide age range, from participants in their early 40 s to a participant aged 67, with only three participants aged 65 or older. While this heterogeneity across life stages is a genuine limitation, it reflects the theoretical argument of the study rather than a sampling error: the 40+ threshold was adopted based on cumulative disadvantage frameworks that locate the onset of aging-related health burden earlier in transgender lives than standard gerontological definitions would suggest [6,7,16,17].

Another issue is that focus group data reflect what participants were willing and able to discuss in a group setting, and experiences of particular sensitivity may not have been raised in that context. The focus groups explored broad healthcare experiences rather than telehealth as a discrete topic. Telehealth emerged organically across all four sessions as a recurring theme of participant-identified concern, and the telehealth implications developed in this paper are derived analytically from those healthcare narratives rather than from telehealth-specific protocols. This reflects the paper’s analytic argument that telehealth cannot be understood apart from the broader healthcare conditions it enters, but future research using telehealth-specific interview designs would allow for a more direct examination of virtual care encounters.

Future research would benefit from dedicated attention to the oldest segments of this population, including participants aged 65 and older whose experiences of transgender aging across longer historical arcs may differ substantially from those of midlife participants.

## 7. Conclusions

The accounts of transmasculine and gender-nonconforming older adults in this study suggest that, without structural transformation, telehealth risks reproducing and amplifying the very harms these participants already navigate. Telehealth is frequently positioned as the solution to barriers in healthcare access for older adults. This study demonstrates that for transgender older adults, telehealth without structural transformation will not solve, and may amplify, existing harms. Provider incompetence, misgendering, inadequate clinical knowledge of aging trans bodies, and disconnection from community care networks are not barriers that technology alone resolves. Addressing these barriers requires a sustained structural commitment: the transformation of provider training, EHR systems, insurance policies, and the fundamental relational orientation of clinical encounters.

This manuscript introduces the Campfire Model of relational telehealth as a conceptual contribution to gerontology, LGBTQ+ health, and telehealth design. Grounded in participant wisdom and consistent with ethics of care, person-centered practice, social connectedness research, and intersectional analysis, the Campfire Model proposes five organizing principles: gathering, warmth, collective knowledge, safety, and accountability. The first four describe the relational and design conditions of affirming care; the fifth names what institutions, insurers, and researchers owe this population before any of the first four can be fully realized.

As the population ages and telehealth expands, the design choices made now will determine whether telehealth becomes a tool for justice or a vehicle for deepening inequities. Transgender older adults deserve healthcare systems designed with and for them: systems that honor their dignity, affirm their identities, recognize their expertise, support their community care infrastructure, and center their own visionary thinking. The Campfire already exists, built by participants in the margins of systems that did not support it. The task before providers, policymakers, and researchers is to resource it, protect it, and ensure that every transgender older adult can find their way to its warmth.

## Data Availability

The original contributions presented in this study are included in the article. Further inquiries can be directed to the corresponding author due to concerns about participant confidentiality.

## Appendix A

**Table A1:** Focus group themes, sub-themes, exemplar quotes, and Campfire Model pillars.

Theme	Key Sub-Themes	Exemplar Quotes	Campfire Model Pillar
**Theme 1** **Provider Competency Crisis** Being patient and educator simultaneously	· Trans 101 required at every new encounter · Providers unprepared for long-term testosterone sequelae · Telehealth scales incompetence · Provider ignorance drives avoidance	“I have to give Trans 101 every time I walk into a new office. I just want someone to know what they’re doing.” **— Participant FD, FG1** “They connected me to someone who had never heard of polycythemia in trans men. I could have been anywhere for that.” **— Participant DS, FG1** “Sometimes I don’t go because I just don’t have enough energy.” **— Participant DS, FG1**	**Safety & Accountability** Mandatory longitudinal competency training required; expanded access without competency delivers harm at scale.
**Theme 2** **Administrative Violence** Architecture of misgendering	· EHR binary architecture misgenders automatically · Accumulation across systems in a single week · Portal abandonment leads to missed diagnoses · Structural erasure distinct from overt hostility	“I have asked them to change it four times. It never changes. After a while, you just feel like the system is telling you something.” **— Participant ER, FG3** “The form just doesn’t have a box for me. That erasure is its own kind of violence.” **— Participant KH, FG2** “I stopped using the portal for two months. And I missed a test result. That’s not a minor problem.” **— Participant WS, FG4**	**Safety** Gender-affirming EHR design is a clinical prerequisite, not a courtesy. Misgendering is a direct harm pathway to care avoidance.
**Theme 3** **Insurance & Political Precarity** Access as fragile political achievement	· Coverage one electoral cycle from elimination · Insurance loss mid-surgery causes iatrogenic harm · Clinical decisions shaped by provider political risk · Telehealth access contingent on legislative stability	“I think about that every election. One elected official could take that away.” **— Participant GF, FG3** “I got stuck halfway through a surgical process. That’s not just inconvenient. That’s dangerous.” **— Participant DS, FG3** “He was managing his risk. With my body.” **— Participant SD, FG3**	**Accountability** Coverage must be structurally insulated from political volatility. No platform redesign resolves this.
**Theme 4** **Aging Transmasculine Body** Uncharted clinical territory	· Shift from transition to aging concerns after 15–30 yrs on T · No protocols for cardiovascular, bone density, or dose adjustment · Polycythemia continues to surprise providers · Peer networks outperform clinical guidance	“Now I’m concerned about what fifteen years of hormones have done in a body that is also just aging. My doctor doesn’t have answers to either question.” **— Participant DS, FG1** “The basics aren’t even what I need anymore. I need someone who knows about long-term cardiovascular effects and dose adjustments as you age.” **— Participant WS, FG4** “I asked this question and was told they would get back to me. That was eight months ago.” **— Participant WS, FG4**	**Accountability** Dedicated longitudinal research investment required. Telehealth cannot deliver competent care until the evidence base exists.
**Theme 5** **The Campfire Vision** *Toward relational telehealth*	· Campfire metaphor introduced spontaneously · Peer networks as primary health knowledge source · Safety defined as continuity and recognition · Virtual formats named as accessible and desired	*“Somewhere you go to be warm, to be with people who know what you’re talking about without explanation, to feel safe. And you can come back.”* **— Participant SD, FG3** *“I found out more about my own health from people in online trans communities than from any doctor. That would be something real.”* **— Participant DS, FG1** *“I want telehealth to be a place I can go that knows me. Not a waiting room I have to convince every time.”* **— Participant CX, FG4**	**Gathering, Warmth & Collective Knowledge** *This theme generates the Campfire Model. Participants envision a relational ecosystem centered on community, peer expertise, and longitudinal continuity.*

***Note.** Themes derived from reflexive thematic analysis of four focus group transcripts (n = 14). Quotes are verbatim from de-identified transcripts; names are pseudonyms. Campfire Model pillars—Gathering, Warmth, Collective Knowledge, Safety, Accountability—are derived from Theme 5. FG = focus group.*
